# Case of Chronic Peritonitis Modified by Scrofula

**Published:** 1854-02

**Authors:** H. M. T. Smith

**Affiliations:** Dunkirk


ART. V.—*Case of Chronic Peritonitis modified by Scrofula.* By H. M. T. SMITH, M. D., Dunkirk.

I send you the following sketch of a recent case, which may be of benefit to the profession:

*Dec.* 18, 1853. Called to see Miss O. R., girl aged 9½ years, dark hair, eyes, and complexion; countenance dull, listless, inexpressive; tongue coated with a thin whitish fur; pulse 90, irritable; appetite invariably poor, over-eating causing severe pain in the stomach and bowels. These attacks are often brought on by exercise, exposure to cold and wet. Epigastrium tender and full; abdomen excessively tender throughout its whole extent. This tenderness is of four years' standing. The bowels are obstinately costive, requiring active cathartics to effect a movement. The abdomen has a rigid feel, as if the peritoneum was too small for the support of the abdominal muscles. The case was originally treated as a case of acute peritonitis, very improperly, with active cathartics.

Gave an anodyne to allay the present paroxysms of distress. Ordered a pill of 2 grs. aloes, ¼ gr. ipecac., 1 gr. rhei, 1 gr. Cayenne pepper. To take 1 pill every four hours till a movement was effected, and, afterward, to take just enough of the pills to keep the bowels regular. These pills were given to determine how far the abdominal tenderness was dependent on functional derangement of the digestive organs. The costiveness was relieved, but the abdomen remains as full and tender as ever.

*Dec.* 20. Ordered the entire abdomen to be painted with strong tincture of iodine. Put it on with a paint brush.

*Dec.* 22. A nice crop of pustules; countenance brightening, as the costiveness is disposed to yield. Suspend the pills and give a fig occasionally. Abdominal tenderness less.

*Dec.* 26. Abdominal tenderness wholly relieved, will bear firm pressure on any part without any pain. Countenance bright, intelligent; appetite good, better than for four years past. Bowels move once a day. Fullness of abdomen diminishing.

Continue iodine externally.

*Dec.* 30. Patient apparently cured. Abdomen has shrunk to its natural size, and in comfort and personal appearance, the girl is well. Indeed the change every way is so marked that one would hardly recognize the girl; is preparing for a New Year's party.

In my humble opinion this is a case of chronic peritonitis modified in its character by a scrofulous tendency and habit. Had the inflammation involved the mesenteric glands at its commencement, the girl would have been emaciated, and perhaps, have died of marasmus. The iodine has done good in two ways, by counter-irritation and by its specific effect. This cannot be a case of simulated peritonitis, described by Sir Benj. Brodie, as the pain was real, of long standing, and was full as severe when pressure was made without the patient's knowledge, as with it.

